# Efficacy of Endoscopic Submucosal Dissection for Superficial Esophageal Cancer on the Distal Side of Esophageal Strictures Using a Novel Thin Therapeutic Endoscope

**DOI:** 10.1002/deo2.70300

**Published:** 2026-04-06

**Authors:** Erika Uchida, Keiichiro Nakajo, Hiroki Yamashita, Atsushi Inaba, Hironori Sunakawa, Tomohiro Kadota, Kensuke Shinmura, Tomonori Yano

**Affiliations:** ^1^ Department of Gastroenterology and Endoscopy National Cancer Center East Chiba Japan; ^2^ Course of Advanced Clinical Research of Cancer Juntendo University Graduate School of Medicine Tokyo Japan; ^3^ Division of Gastroenterology and Hepatology, National Kohnodai Medical Center Chiba Japan; ^4^ Medical Device Innovation Center National Cancer Center Hospital East Chiba Japan

**Keywords:** endoscopic submucosal dissection, esophageal dilation, esophageal squamous cell carcinomas, stricture, therapeutic thin endoscope

## Abstract

**Objectives:**

Endoscopic submucosal dissection (ESD) for esophageal squamous cell carcinomas (ESCCs) localized on the distal side of strictures requires dilation to pass a conventional endoscope. While an ultra‐slim endoscope can pass without dilation, ESD using it remains clinically challenging. Despite its small outer diameter of only 7.9 mm, the EG‐840TP features a 3.2‐mm working channel, an auxiliary water channel, and 160° downward angulation capability. We evaluated the efficacy and safety of ESD using the EG‐840TP for ESCCs localized on the distal side of strictures.

**Methods:**

This retrospective study included patients who underwent ESD for ESCCs localized on the distal side of strictures that a conventional endoscope (diameter ≥8.9 mm) could not pass at our institute from December 2015 to November 2024. Patients were categorized into novel thin endoscope (the EG‐840TP) and conventional endoscope (treated after stricture dilation) groups. Patients with strictures narrower than 6 mm were excluded.

**Results:**

Of 28 patients (36 lesions), 13 patients (19 lesions) underwent ESD using the novel thin endoscope, whereas 15 patients (17 lesions) underwent ESD using the conventional endoscope. The treatment speed was significantly faster in the novel thin endoscope group (4.91 mm^2^/min vs. 1.63 mm^2^/min, *p* = 0.03). No serious adverse events were observed in either group.

**Conclusions:**

ESD using the EG‐840TP demonstrated superior efficacy for superficial ESCCs located on the distal side of strictures, offering an alternative to dilation‐based approaches.

## 1 Introduction

Effective definitive treatments for esophageal cancer, including surgery, endoscopic submucosal dissection (ESD), and chemoradiotherapy (CRT), sometimes lead to refractory benign esophageal strictures. [1, 2, 3, 4]. Esophageal, laryngeal, and pharyngeal cancers have a high risk of metachronous recurrence, and regular endoscopic surveillance is recommended following definitive treatment. When metachronous recurrent lesions are detected and diagnosed as superficial esophageal squamous cell carcinomas (ESCCs), they are typically indicated for ESD [5, 6, 7, 8, 9, 10]. Definitive treatments for ESCCs sometimes lead to refractory benign esophageal strictures; in many cases, therapeutic endoscopes cannot allow passage through strictures. In such instances, ESD for ESCCs located distal to the stricture is commonly performed after dilation using methods such as endoscopic balloon dilation (EBD) or the radial incision and cutting (RIC) technique [11, 12, 13, 14, 15, 16, 17, 18, 19].

Dilation carries risks, such as bleeding and perforation, and excessive dilation is restricted for safety. It is difficult for a therapeutic endoscope with a cap to pass through strictures [11]. Conversely, a thin endoscope can pass through strictures without dilation [20, 21], and cases of ESD using thin endoscopes have been reported [22, 23]. However, the functionality and maneuverability of thin endoscopes are limited compared to those of conventional endoscopes, highlighting clinical challenges [24, 25].

**FIGURE 1:**
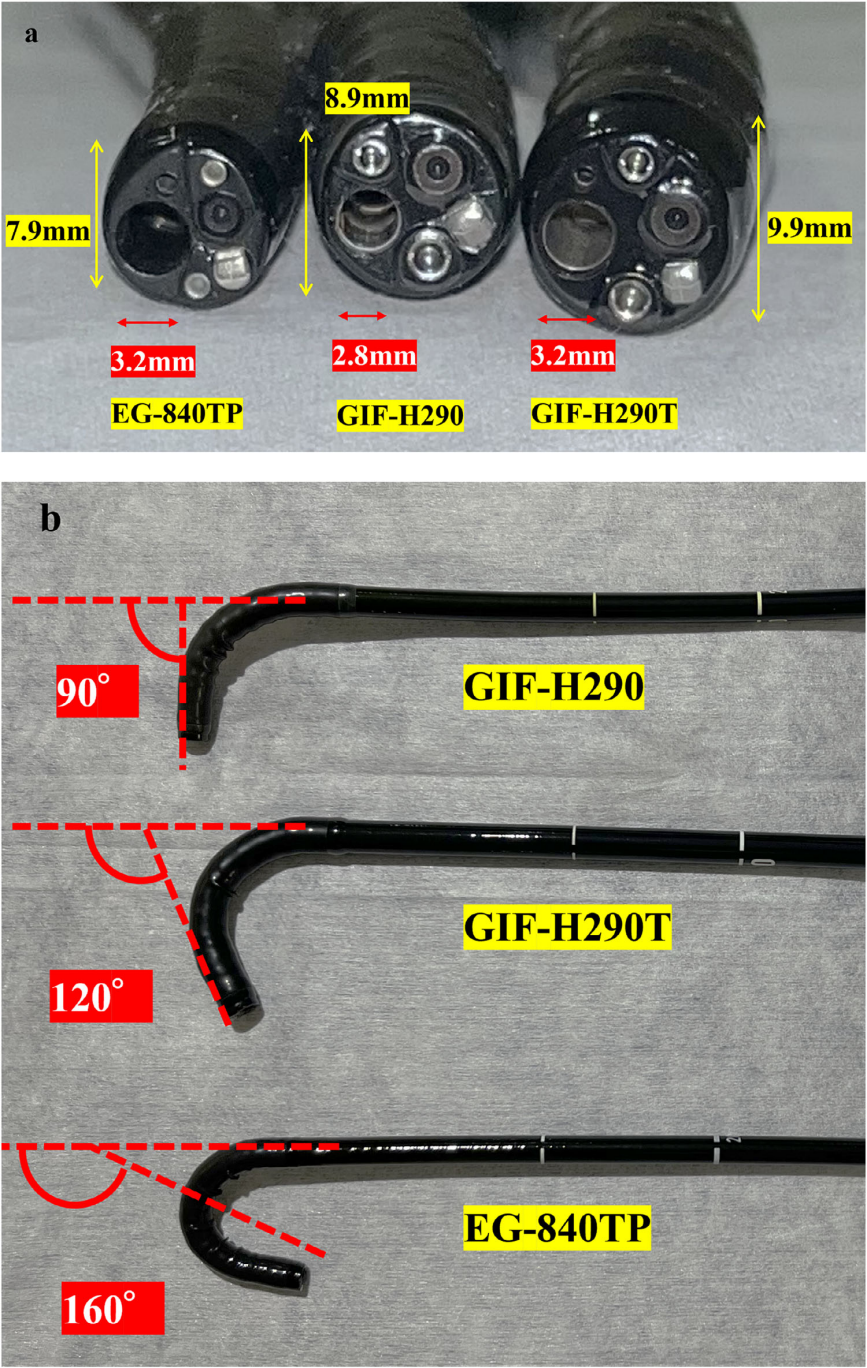
Comparison of the features of the EG‐840TP with the GIF‐H290 and GIF‐H290T. (a) Comparison of the outer diameter and channel inner diameter of the EG‐840TP compared to the GIF‐H290 and GIF‐H290T endoscopes. (b) Comparison of the down angulation ranges of the EG‐840TP, GIF‐H290, and GIF‐H290T. These pictures show the actual novel thin therapeutic endoscope (EG‐840TP) alongside the GIF‐H290 (most frequently used in the conventional endoscope group) and the GIF‐H290T (commonly used for standard ESD). The distal end outer diameter of the EG‐840TP is 7.9 mm, compared to 8.9 mm for the GIF‐H290. The channel inner diameter is 3.2 mm, compared to 2.8 mm for the GIF‐H290. The EG‐840TP features an auxiliary water channel, which irrigates water through the inlet when necessary (e.g., in cases of severe bleeding). Additionally, the down angulation range of the EG‐840TP is 160°, compared to 90° for the GIF‐H290. For reference, the features of the GIF‐H290T are a distal end outer diameter of 9.8 mm, a channel inner diameter of 3.2 mm, an auxiliary water channel, and a down angulation range of 120°.

A novel thin therapeutic endoscope (EG‐840TP; Fujifilm Corp., Tokyo, Japan) with an outer diameter of 7.9 mm was recently released. This endoscope features a 3.2 mm working channel, an auxiliary water channel, and 160° downward angulation capability, offering enhanced performance compared to conventional therapeutic endoscopes. Its smaller distal‐end diameter may facilitate ESD without dilation in cases with strictures, which is an effective technique (Figure 1). Although there have been case reports demonstrating the efficacy of the EG‐840TP for ESD [26, 27, 28, 29, 30], the thin endoscope may have certain limitations in maneuverability because of its narrower outer diameter. Furthermore, the efficacy and safety of ESD using this novel thin therapeutic endoscope in cases with strictures have not been fully evaluated.

The aim of this study was to assess the efficacy and safety of ESD using the novel thin endoscope for metachronous recurrence of superficial ESCCs localized on the distal side of strictures that could not be passed through by a conventional endoscope, compared to ESD using a conventional endoscope after dilation.

## 2 Methods

### 2.1 Study Design

This retrospective, single‐center observational study was conducted at the National Cancer Center Hospital East, Kashiwa, Japan. We analyzed patients who underwent ESD for ESCCs localized on the distal side of strictures that could not be passed by a conventional endoscope (≥8.9 mm in diameter) at our institute from December 2015 to November 2024.

Patients who underwent ESD using a GIF‐1200N (Olympus; hereafter, “the ultra‐slim endoscope”), having a 2.2‐mm channel, or EG‐L580NW (Fujifilm) were excluded. Patients with strictures <6 mm in diameter (which could allow the passage of GIF‐1200N, GIF‐XP290N, or EGL580NW; 5.8 mm outer diameter) were also excluded to ensure comparability between the two groups, as such cases were not treated using the novel thin endoscope.

Patients were categorized into two groups: the novel thin endoscope group (treated using EG‐840TP) and the conventional endoscope group (treated using a conventional endoscope ≥8.9 mm in diameter). Treatment outcomes were evaluated and compared between the groups.

### 2.2 Endoscopic Strategy, Devices, and Indications for ESD

EG‐840TP was introduced at our institution in June 2023. Before its introduction, the strategy for passing strictures involved attempting passage with a conventional endoscope with a cap after dilation. If the passage was unsuccessful, the cap was removed, and another attempt was made. When the endoscope still could not pass, an ultra‐slim endoscope was used. After June 2023, initial attempts to pass the stricture were generally made using EG‐840TP.

In the novel thin endoscope group, DH‐083ST (Fujifilm Corporation) was used as a transparent cap. In the conventional endoscope group, the Elastic Touch slit and hole type S (Top Corporation, Tokyo, Japan) was used. In the conventional group, GIF‐Q260, GIF‐H290, or GIF‐Q260J (Olympus Corporation) (Table 1) was used, based on the operator's discretion. To allow passage of a conventional endoscope during treatment, dilation was performed using either EBD or RIC before the procedure. RIC was primarily selected for cases with either a prior history of RIC or multiple failed EBD procedures. However, the method of dilation was ultimately determined at the discretion of the operators based on the degree of stricture. The criteria for indicating ESD for ESCC were (1) depth of invasion of the lesion being limited to within MM/SM1, (2) confirmation via computed tomography scan that there was no lymph node or distant metastasis if the depth of invasion exceeded clinically MM, (3) histologically confirmed squamous cell carcinoma with biopsy specimens prior to ESD, and (4) lesions without deep ulcers.

**TABLE 1:** Features of the endoscope and number of patients.

	EG‐840TP	GIF‐Q260	GIF‐H290	GIF‐Q260J	GIF‐H290T
Outer diameter (mm)	7.9	8.9	9.2	9.9	9.8
Working channel size (mm)	3.2	2.8	2.8	3.2	3.2

An auxiliary water channel	Present	Absent	Absent	Present	Present
Downward angulation Capability (°)	160	90	90	90	120
The number of patients	19	8	8	1	0

### 2.3 Radial Incision and Cutting

RIC procedures were performed as previously described, using either IT Knife‐2 (KD‐611L; Olympus Corporation) or IT Knife Nano (KD‐612; Olympus Corporation) [11]. First, the stricture was incised radially. Next, an imaginary line connecting the esophageal lumen on the oral and anal sides was assumed, along which the incision was extended. This process was repeated until a conventional endoscope with a transparent cap could pass. If the passage was unsuccessful, the cap was removed. IT Knife‐2, with its insulated tip and three sharp blades, enables precise incisions, while IT Knife Nano, with its smaller tip, facilitates passage through severe strictures, reducing the risk of deep incisions and perforation. The choice of knife was at the operator's discretion.

### 2.4 Endoscopic Balloon Dilation

After assessing the stricture, dilation was performed using either the CRE balloon (Boston Scientific Corporation, Marlborough, MA, USA) or the E‐dive balloon (Nipro, Tokyo, Japan). Since the outer diameter of the GIF‐Q260 (9.2 mm) with the Elastic Touch slit and hole‐type S has a total diameter of ≥12 mm, balloon sizes of 12–15 mm or larger were used. The EBD procedure involved (1) positioning the endoscope near the stricture and inserting an appropriate balloon catheter, (2) inflating the balloon while monitoring pressure with the Alliance II system (Boston Scientific), and (3) advancing a conventional endoscope (GIF‐H290, GIF‐Q260, or GIF‐Q260J) past the dilated stricture. If passage with a transparent cap was unsuccessful, the cap was removed, and passage of the endoscope was attempted again.

### 2.5 Endoscopic Submucosal Dissection

Previously, midazolam and dexmedetomidine were used for sedation; however, recently, these have been replaced by flunitrazepam and dexmedetomidine during ESD, and the dosage was adjusted based on the patient's age, background, and other factors. Marking dots were placed around the lesion margins using DualKnife (KD‐650Q; Olympus Corporation). A solution of 0.4% sodium chloride mixed with hyaluronic acid, epinephrine, and indigo carmine was injected into the submucosal layer. The incision and trimming were then performed using DualKnife. After the circumferential incision, traction was applied using a clip at the operator's discretion, followed by submucosal dissection and resection. If the operator judged that it would be difficult to complete the procedure using ESD only, snaring was performed after the circumferential incision.

### 2.6 Assessment of ESD Efficacy, Safety, and Factors Associated With Treatment Speed

The efficacy was evaluated based on procedural success, procedure time (for ESD and ESD+ dilation), and treatment speed. The procedural success was defined as completion of the procedure using ESD only. The procedure time for ESD was defined as the interval from the marking of the tumor's periphery to completion of the resection. The dilation procedure time was defined as the time from the insertion of the dilation instrument through the endoscope until successful passage of the endoscope through the stricture. The procedure time for cases in which a snare was used in combination was defined as the duration from marking until the specimen was completely resected by the snare. The tumor was assumed to be an ellipse, and the area was calculated as: lesion area = Long diameter × Short diameter ×π/4. The treatment speed was then determined by dividing the lesion area by the procedure time; the same calculation was applied when a snare was used. In cases of synchronous lesions, the treatment time and tumor diameter were recorded separately for each lesion. For patients with two lesions, the marking and procedure times for each individual lesion were determined by reviewing the intraoperative video recordings. Factors associated with treatment speed were further evaluated using both univariate and multivariable analyses by selecting candidate variables based on previous studies and clinical relevance. The distances from the incisors to the stricture and to the ESCC lesion were recorded during each endoscopic examination, and the distance between the stricture and the lesion was then retrospectively calculated as the difference between these two values. Safety was evaluated based on severe adverse events (post‐ESD bleeding and perforation).

### 2.7 Histopathological Assessments

Each resected specimen was fixed in formalin with extension, cut into 2–3 mm sections, and sectioned at 2 µm. Sectioned resected specimens were mounted on glass slides, stained with hematoxylin and eosin, and evaluated in accordance with the Japanese Classification of Esophageal Cancer. Final diagnoses were made by a qualified pathologist. Histopathological assessment was considered challenging in cases where large areas of the esophageal epithelium had detached and where the thermal cautery effect had denatured the subepithelial tissue.

### 2.8 Statistical Analysis

Categorical variables were compared using Fisher's exact test, and continuous variables using the t‐test. Continuous variables are presented as means with standard deviations or as medians with ranges. To evaluate associations with treatment speed, ROC curve‐derived optimal cut‐off values were used to dichotomize age, lesion area, and distance from the stricture. Factors with *p* < 0.05 in univariate analysis were entered into a multiple regression model to identify independent predictors of treatment speed. Statistical significance was set at *p* < 0.05. All analyses were performed using EZR (Saitama Medical Center, Jichi Medical University, Saitama, Japan).

## 3 Results

### 3.1 Patient Flow and Characteristics of Patients’ Lesion and Stricture

**FIGURE 2:**
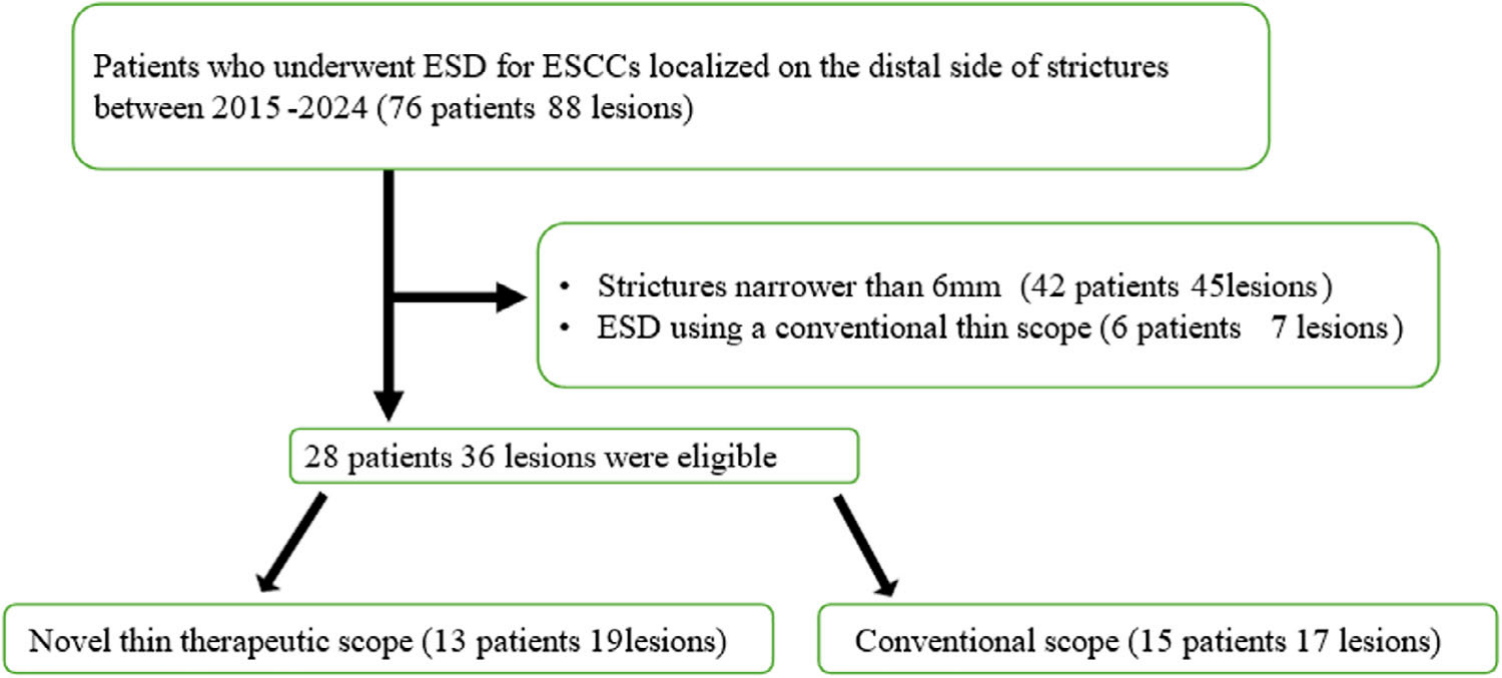
Patient participation flow. A total of 76 patients (88 lesions) who underwent ESD for superficial ESCCs localized distal to esophageal strictures were identified. During the study period, 42 patients (45 lesions) were excluded due to strictures narrower than 6 mm, and six patients (seven lesions) were excluded because ESD was performed using the ultra‐slim endoscope. Ultimately, 28 patients with 36 lesions were included in the analysis. The novel thin endoscope group comprised 13 patients (19 lesions), while the conventional endoscope group comprised 15 patients (17 lesions).

During the study period, we retrospectively investigated 76 patients with 88 lesions who underwent ESD for ESCCs localized on the distal side of strictures caused by previous treatment for esophageal, laryngeal, or pharyngeal cancer, or by the tumor itself. Forty‐eight patients were excluded: 42 patients (45 lesions) due to severe strictures with a diameter <6 mm, and six patients (seven lesions) because ESD was performed using the ultra‐slim endoscope. Finally, 28 patients with 36 lesions were included. Patients were divided into the novel thin endoscope (13 patients, 19 lesions) and the conventional endoscope (15 patients, 17 lesions) groups (Figure 2).

Table 2 summarizes patient characteristics. Tumor size was slightly larger in the novel thin group (*p* = 0.31). Causes of strictures differed significantly between the groups (*p* = 0.04), with CRT‐induced strictures being more frequent in the thin group (Table 3).

**TABLE 2:** Baseline patients and lesion characteristics.

	Novel thin endoscope	Conventional endoscope	*p*‐Value
No. of patient	13	15	
Sex			
Male	9 (69.2%)	11 (73.3%)	1
Female	4 (30.8%)	4 (26.7%)	
Age, years, median (range)	66 (46–79)	68 (54–88)	0.10
No. of lesions	19	17	
Operator			
Experienced	15 (78.9%)	11 (64.7%)	0.46
Resident	4 (21.1%)	6 (35.3%)	
Tumor location			
Upper (Ce, Ut)	10 (52.6%)	9 (53.0%)	0.68
Middle (Mt)	7 (36.8%)	4 (23.5%)	
Lower (Lt, Ae)	2 (10.6%)	4 (23.5%)	
Tumor size (mm), mean ± SD	15.4 (±15.4)	11 (±7.85)	0.31
Destination between stricture and tumor, mean ± SD	5.3 (±5.2)	5.8 (±5.0)	0.77
Scar close to the lesions	12 (63.2%)	11 (64.7%)	1
Lugol‐voiding lesions			
Grade A	0	0	0.47
Grade B	0	1 (5.9%)	
Grade C	19 (100%)	16 (94.1%)	
Circumferential of the tumor			
<1/4	12 (63.2%)	16 (94.1%)	0.04
≥1/4	7 (36.8%)	1 (5.9%)	

Abbreviations: Ae, abdominal esophagus; Ce, cervical; Lt, lower thoracic; Mt, middle thoracic; SD, standard deviation; Ut, upper thoracic.

**TABLE 3:** Baseline characteristics of the strictures.

	Novel thin endoscope (*n* = 19)	Conventional endoscope (*n* = 17)	*p*‐Value
Stricture location			
Pharynx and larynx	11 (57.9%)	7 (41.2%)	0.40
Upper (Ce, Ut)	7 (36.8%)	7 (41.2%)	
Middle (Mt)	1 (5.3%)	3 (17.6%)	
Cause of stricture			
Endoscopic treatment	4 (21.1%)	11 (64.7%)	0.04
Chemoradiotherapy	7 (36.8%)	4 (23.5%)	
Surgical treatment	7 (36.8%)	2 (11.8%)	
Tumor	1 (5.3%)	0	
Number of EBD sessions			
0	8 (42.0%)	6 (35.4%)	0.97
1–5	4 (21.1%)	4 (23.5%)	
6–10	3 (15.8%)	4 (23.5%)	
≥11	4 (21.1%)	3 (17.6%)	
Number of RIC sessions			
0	16 (84.2%)	17 (100%)	0.23
≥1	3 (15.8%)	0	

Abbreviations: EBD, endoscopic balloon dilation; Ce, cervical; Mt, middle thoracic; RIC, radial incision and cutting; Ut, upper thoracic.

### 3.2 ESD Outcomes and Factors Associated With Treatment Speed

Table 4 summarizes ESD outcomes. The procedural success rate was non‐significantly higher in the novel thin endoscope group than in the conventional endoscope group (94.7% vs. 88.2%; *p* = 0.59). In two cases in the conventional endoscope group and one in the novel thin endoscope group, the procedure could not be completed using ESD only. However, these procedures were successfully completed using snaring. The mean procedure time did not differ significantly between the groups (38.1 ± 40.3 vs. 41.9 ± 33.9 min; *p* = 0.76). However, treatment speed was significantly faster in the novel thin endoscope group (4.91 mm^2^/min vs. 1.63 mm^2^/min; *p* = 0.03). All lesions were resected en bloc. The passage success rate of the endoscope with a cap was significantly higher in the novel thin endoscope group than in the conventional endoscope group (89.5% vs. 52.9%; *p* = 0.03). No serious adverse events were observed in either group (Table 4).

**TABLE 4:** Outcomes of endoscopic balloon dilation (ESD) and dilation.

	Novel thin endoscope (*n* = 19)	Conventional endoscope (*n* = 17)	*p*‐Value
Procedural success			
ESD completed	18 (94.7%)	15 (88.2%)	0.59
With snaring	1(5.3%)	2 (11.8%)	
Procedure time^a^ (min), mean ± SD			
ESD	38.1 (±40.3)	41.9 (±33.9)	0.76
Dilation + ESD	38.1 (±40.3)^c^	47.5 (±34.4)	0.46
Treatment speed (mm^b^/min), mean ± SD	4.9 (±5.7)	1.6 (±1.6)	0.03
Passage success rate of the endoscope with a cap	17 (89.5%)	9 (52.9%)	0.03
ESD with the traction method	13 (68.4%)	7 (41.2%)	0.17
ESD with dilation	0	17 (100%)	<0.01
*En bloc* resection	19 (100%)	17 (100%)	1
Adverse events			
Bleeding^b^	0	0	—
Perforation	0	0	—

Abbreviations: ESD, endoscopic submucosal dissection; SD, standard deviation.

^a^ Procedure time of ESD was defined as the interval between marking the tumor's periphery and the completion of resection.

^b^ Bleeding was defined as bleeding within the first 30 days meeting any of the following criteria: (1) hematemesis or melena, (2) >2 g/dL drop in hemoglobin, or (3) endoscopic, radiologic, or surgical procedure because of suspicion of bleeding.

^c^ No dilatation was performed in the novel thin endoscope group.

In the univariate analysis, endoscope group, distance from stricture, and lesion area had *p* < 0.05. In the multivariate analysis, all three factors remained significantly and independently associated with treatment speed (Table 5).

**TABLE 5:** Univariate and multivariate analysis of factors independently associated with resection speed.

	Univariate analysis		Multivariate analysis	
**Subgroup**	**Estimated Coefficient** **(95% CI)**	***p*‐Value**	**Estimated Coefficient** **(95% CI)**	***p*‐Value**
Endoscope group				
Novel thin	3.28 (0.27–6.28)	0.03	2.86 (0.77–4.95)	<0.01
Conventional	Ref.	—	Ref.	—
Lesion area				
Small area (<56 mm^2^)	5.69 (3.14–8.24)	<0.01	3.88 (1.49–6.27)	<0.01
Large area (≥56 mm^2^)	Ref.	—	Ref.	—
Distance from stricture				
Near (<10 cm)	6.56 (3.35–9.77)	<0.01	4.23 (1.33–7.14)	<0.01
Far (≥10 cm)	Ref.	—	Ref.	—
Lesion near scar				
Yes	−2.14 (‐5.42–1.14)	0.19		
No	Ref.	—		
Expert				
Resident	−3.14 (−6.49–0.21)	0.07		
Expert	Ref.	—		
Age (years)				
≤65	1.67 (−1.54–4.89)	0.30		
≥66	Ref.	—		
Sex				
Male	−0.17 (‐3.54–3.20)	0.92		
Female	Ref.	—		
Use of a traction device				
Without traction	−1.26 (−1.47–1.96)	0.43		
With traction	Ref.	—		

Abbreviation: CI, Confidence interval.

### 3.3 Pathological Outcome

Table 6 presents pathological outcomes. One lesion (5.9%) in the conventional endoscope group could not be evaluated pathologically due to specimen damage caused by technical difficulty during the procedure. Negative vertical margins were achieved in 15 lesions (83.3%) in the novel thin endoscope group and 15 lesions (93.8%) in the conventional endoscope group (*p* = 1.0). Negative horizontal margins were observed in 10 lesions (55.5%) in the novel thin endoscope group and 13 lesions (81.3%) in the conventional endoscope group (*p* = 0.20). The rate of positive horizontal margins was significantly higher in lesions with a circumferential extent greater than one‐quarter compared to those with an extent of less than one‐quarter. The depth of invasion was generally within the indications for endoscopic treatment; however, one lesion showed submucosal invasion (SM2). Although the vertical margin was positive, the lesion was resected en bloc without snaring (Table 6).

**TABLE 6:** Pathological outcomes study design, definitions, and ethical considerations.

	Novel thin endoscope (*n* = 19)	Conventional endoscope (*n* = 17)	*p*‐Value
Depth of invasion			
EP	12 (66.7%)	15 (93.8%)	0.04
LPM	5 (27.8%)	0	
MM	0	1 (6.2%)	
SM2	1 (5.5%)	0	
Vascular invasion			
Negative	17 (94.4%)	16 (100%)	1
Positive	1 (5.6%)	0	
Lymphatic invasion			
Negative	18 (100%)	16 (100%)	1
Positive	0	0	
Vertical margin			
Negative	15 (83.3%)	15 (93.8)	1
Positive	1 (5.6%)	0	
Indeterminate	2 (11.1%)	1 (6.2%)	
Horizontal margin			
Negative	10 (55.5%)	13 (81.3%)	0.20
Positive	5 (27.8%)	1 (6.2%)	
Indeterminate	3 (16.7%)	2 (12.5%)	
Not evaluable	1 (5.3%)	1 (5.9%)	1

## 4 Discussion

This study revealed that ESD using the novel thin therapeutic endoscope can be performed effectively and safely for superficial ESCCs localized on the distal side of strictures. With the novel thin endoscope, the stricture could be passed with the cap attached without dilation, submucosal dissection could be performed more rapidly, and despite its small diameter, maneuverability was not compromised. Ultra‐slim endoscopes, due to their small outer diameter and soft shaft, have limitations in maneuverability, particularly the direct transmission of rotational movements from the right hand to the endoscope tip, which hinders precise control. This is primarily attributed to insufficient shaft stiffness, limiting its maneuverability [25, 26]. Although the novel thin endoscope is not as slim as the conventional one, it remains considerably smaller in diameter, raising initial concerns regarding its maneuverability during ESD procedures. However, the present study demonstrated that the treatment speed was significantly faster in the novel thin endoscope group compared to the conventional endoscope group. ESD for entire circumferential lesions was successfully completed without issues in the novel thin endoscope group. Three factors may have contributed to this improvement. First, the shaft stiffness of the novel thin endoscope was superior to that of the ultra‐slim endoscope, potentially contributing to the improved treatment speed. The novel thin endoscope shaft exhibits a gradual change in stiffness and elasticity from the flexible shaft to the handgrip, allowing the tip to maintain flexibility while increasing stiffness near the handgrip, improving maneuverability despite its smaller diameter. Second, the passage success rate of the endoscope with a cap was significantly higher in the novel thin endoscope group. The transparent cap at the tip of the endoscope likely enhanced the visibility of the submucosal layer, facilitating faster dissection [31, 32]. Third, the ultra‐slim endoscope was limited by a narrow working channel, which hindered the use of traction devices and performed sufficient suction during bleeding. Furthermore, the lack of an auxiliary water channel prevented irrigation with a water jet. The novel thin therapeutic endoscope addressed these issues with a widened working channel, allowing the insertion and removal of traction devices (e.g., clip appliers), improving suction efficiency. Furthermore, it was equipped with an auxiliary water channel, enabling irrigation during bleeding. Thus, ESD using the novel thin therapeutic endoscope achieves comparable efficacy and safety to procedures using conventional endoscopes.

The rate of lesions with a negative horizontal margin was lower in the novel thin endoscope group than in the conventional endoscope group. This may be because lesions in the novel thin endoscope group tended to have a greater circumferential extent. In clinical practice, to reduce the risk of post‐ESD stenosis or technical difficulties [3, 4, 33], marking dots are often placed very close to the lesion border in circumferentially wide lesions. Additionally, if the novel thin endoscope group included a high rate of cases with lesions close to strictures or scars, or if iodine‐unstained regions of the esophageal mucosa (LVL Grade C) made it difficult to delineate lesion borders, the rate of positive horizontal margins might have been affected. However, no significant differences were observed between the two groups regarding proximity to strictures or scars, or in the prevalence of LVL Grade C. Therefore, the lower rate of negative horizontal margins is most likely attributable to the greater circumferential extent of the lesions.

This study had several limitations. First, it was a retrospective, single‐center study. Second, patients with strictures narrower than 6 mm were excluded. Third, after the novel thin therapeutic endoscope was released, almost all ESD procedures were performed using the novel thin endoscope, leading to a temporal imbalance between the comparison groups. Fourth, the strictures caused by CRT were generally more rigid and accounted for a higher percentage of the causes. The novel thin therapeutic endoscope group may have been at a disadvantage regarding treatment outcomes. Fifth, the use or nonuse of traction may influence ESD outcomes, including procedural speed. However, no explicit criteria for the application of traction were defined in this study, and the decision was left to the discretion of the operator.

Nevertheless, the findings provide evidence that ESD using the novel thin therapeutic endoscope is effective for superficial ESCCs located distal to strictures, and this approach offers a viable alternative to dilation‐based approaches.

## Author Contributions

**Erika Uchida** curated datasets, performed the formal analysis, created a visualization, and drafted the original manuscript. **Keiichiro Nakajo** designed and provided supervision, acquired funding for the research, and critically revised the manuscript. **Hiroki Yamashita**, **Atsushi Inaba**, **Hironori Sunakawa**, **Tomohiro Kadota**, **Kensuke Shinmura**, and **Tomonori Yano** contributed to manuscript review and editing. **Tomonori Yano**, one of the authors, is a Deputy Editor‐in‐Chief of Digestive Endoscopy. The remaining authors declare no conflicts of interest related to this article.

## Funding

This work was supported by the National Cancer Center research and development fund [grant number 2025‐A‐07].

## Ethics Statement

This retrospective, single‐center observational study was approved by the Institutional Review Board of the National Cancer Center (Approval No. 2017–434) and conducted in accordance with the guidelines for epidemiological studies of the Japan Ministry of Health, Labour and Welfare.

## Consent

Because this was a retrospective study, written informed consent from individual patients was not required. Instead, an opt‐out approach was used, and study information was provided on the hospital website.

## Conflicts of Interest

Tomonori Yano, one of the authors, is a deputy Editor‐in‐Chief of *Digestive Endoscopy*. The remaining authors declare no conflicts of interest.

## Declaration of Generative Artificial Intelligence and Artificial Intelligence‐Assisted Technologies in the Writing Process

No generative artificial intelligence (AI) or AI‐assisted technologies were used in the writing or editing of this manuscript.

## Clinical Trial Registration

N/A.
